# MiR-206 regulates the Th17/Treg ratio during osteoarthritis

**DOI:** 10.1186/s10020-021-00315-1

**Published:** 2021-06-19

**Authors:** Xiguang Ye, Qilin Lu, Aofei Yang, Jun Rao, Wei Xie, Chengjian He, Weijun Wang, Hao Li, Zhiwen Zhang

**Affiliations:** 1 Department of Orthopedics, Hubei Provincial Hospital of Traditional Chinese Medicine, No.4, Hua-Yuan-Shan, Yanzhi Road, Wuchang District, Wuhan, 430061 Hubei China; 2 Institute of Orthopedics, Hubei Province Academy of Traditional Chinese Medicine, No.4, Hua-Yuan-Shan, Yanzhi Road, Wuchang District, Wuhan, 430070 Hubei China; 3 Department of Orthopedics, Hubei 672 Orthopaedics Hospital of Integrated Chinese & Western Medicine, Wuhan, 430079 Hubei China; 4 College of Acupuncture and Orthopedics, Hubei University of Chinese Medicine, No.4, Hua-Yuan-Shan, Yanzhi Road, Wuchang District, Wuhan, 430061 Hubei China

**Keywords:** miR-206, Osteoarthritis, T helper 17 cells, Regulatory T cells

## Abstract

**Background:**

The present study aimed to determine the functional role of miR-206 in T helper 17 (Th17)/regulatory T (Treg) cell differentiation during the development of osteoarthritis (OA).

**Methods:**

Patients with OA and healthy controls were recruited for investigating the association between miR-206 and Th17/Treg ratio. Transfection experiments were conducted in CD4^+^ T cells to verify the mechanism of miR-206 on the balance of Treg/Th17. OA model was constructed to detect the clinical score, histopathological changes and Treg/Th17 ratio. OA model was induced in rats to verify the effect of miR-206 inhibition on Th17/Treg immunoregulation.

**Results:**

High expression of miR-206 was positively correlated with peripheral Th17/Treg imbalance in patients with OA. The interactions between miR-206 and the 3′ untranslated regions (3'-UTR) of suppressor of cytokine signaling-3 (SOCS3) and fork head transcriptional factor 3 (Foxp3) were confirmed by luciferase reporter assays. MiR-206 disturbed the Th17/Treg balance by targeting SOCS3 and Foxp3. In vivo assay demonstrated that antagomiR directed against miR-206 restored Th17/Treg balance during the development of OA.

**Conclusion:**

MiR-206 contributed to the progression of OA by modulating Th17/Treg imbalance, suggesting that miR-206 inhibition might be a promising therapeutic strategy for the treatment of OA.

**Supplementary Information:**

The online version contains supplementary material available at 10.1186/s10020-021-00315-1.

## Background

Osteoarthritis (OA) is a chronic joint disease involving articular cartilage erosion, osteophyte formation, subchondral sclerosis, and the morphological changes in synovium and articular capsule (Pereira et al. 2015). Patients with OA exhibit infiltration of CD4^+^T lymphocytes in synovial tissues (Moradi 2015). Emerging studies suggest that the imbalance between pro-inflammatory T helper 17 (Th17) cells and anti-inflammatory regulatory T (Treg) cells is a key inducement in the pathogenesis of inflammatory diseases, including OA (Li et al. 2017). However, the specific mechanism underlying Th17/Treg balance in the occurrence of OA remains unclear.

The differentiation of Th17 cells involves the activation and phosphorylation of signal transducer and activator of transcription 3 (STAT3) and the downstream regulation of retinoid-related orphan receptor γt (RORγt) (Singh 2014). Suppressor of cytokine signaling-3 (SOCS3) is considered a key signaling molecule that regulates Th17 cell differentiation by negatively regulating STAT3-activating cytokines (Shi 2019). Meanwhile, Treg cells expressing the transcription factor fork head transcriptional factor 3 (Foxp3) have a crucial role in the induction of immunosuppression (Kawai et al. 2018).

MiRNAs participate in many physiological processes, such as cell proliferation, differentiation and apoptosis (Tafrihi and Hasheminasab 2019). Existing studies have reported that some miRNAs play crucial roles in regulating immune balance, especially in regulating Th17/Treg cell differentiation. For example, in experimental autoimmune hepatitis (AIH), antagomir-155 inhibited the differentiation of Th17/Treg by suppressing the secretion of IL-17A and IL-23, thereby alleviating the progression of AIH (Xia et al. 2018). Singh et al. found that two highly associated miRNAs (miR-15b and miR-16) enhanced Th17 differentiation and hindered the function of Treg cells through downregulating IL-9 expression (Singh et al. 2016). A previous study indicated that miR-206 was highly expressed in OA patients, and the overexpression of miR-206 was capable of inhibiting the proliferation of chondrocytes and promoting apoptosis, suggesting that miR-206 may be involved in the occurrence and development of OA (Ni et al. 2018). However, it is unclear whether miR-206 was associated with the imbalance of Th17/Treg cells in the pathogenesis of OA. Our present study aimed to explore miR-206-mediated immune regulation in the pathogenesis of OA.

In the present study, we revealed that miR-206 was consistently upregulated in the PBMCs from OA patients. Subsequently, we found that miR-206 promoted the Th17 differentiation, while inhibiting the differentiation of Treg cells. Furthermore, we validated that SOCS3 and Foxp3 are both direct target genes of miR-206 and confirmed that miR-206 mediates Th17/Treg differentiation by negatively regulating SOCS3 and Foxp3 expression. Thus, our results suggest that miR-206 is a Th17/Treg cell-associated miRNA that functions in the pathogenesis of OA.

## Materials and methods

### Patients

A total of 30 consecutive patients with OA (age range, 23–80 years old; male/female, 18/12) undergoing hip joint replacement in the Department of orthopedics, Hubei Provincial Hospital of Traditional Chinese Medicine between October 2016 and October 2017. Meanwhile, 26 age- and sex-matched healthy people undergoing surgery due to femoral neck fracture were recruited as the control group. All procedures were performed according to the guidelines of the ethical committee of Hubei Provincial Hospital of Traditional Chinese Medicine and all subjects provided informed consent to participate in the study (approval: HBZY2018-C22-01). Demographic data of the OA patients and the control group were shown in Table 1, and no significant statistical differences exist between groups.

**Table 1:** Demographic data of the OA patients and the control group

	OA patients (n = 30)	Control (n = 26)	
Male	18	17	*P* > 0.05
Female	12	9	*P* > 0.05
Age range (years)	28–72	30–65	*P* > 0.05
Average age (years)	48.3 ± 5.7	49.9 ± 6.1	*P* > 0.05

### *Isolation and culture of CD4*^+^*T Cells*

Whole-blood samples (15 ml) were obtained from all of the enrolled subjects. Human peripheral blood mononuclear cells (PBMCs) were isolated from the whole blood samples by Ficoll-Hypaque density gradient centrifugation. After isolation, the PBMCs were washed twice and resuspended in PBS to isolate CD4^+^ T lymphocytes using a magnetic cell sorting method following the manufacturer’s instructions (Invitrogen, Carlsbad, CA, USA).

For Th17 differentiation, CD4^+^ T cells were cultured for 3 days under Th17-cell polarizing conditions: RPMI-1640 medium containing 10% fetal calf serum, 1 mM glutamine, 0.1 mM beta-mercaptoethanol, 1% nonessential amino acids (Sigma-Aldrich, St. Louis, MO, USA), 5 ng/mL IL-2 (R&D Systems, Minneapolis, MN, USA), 20 ng/mL IL-6, 5 ng/mL transforming growth factor-b, 10 ng/mL IL-23, 2 mg/mL antiIL-4, 2 mg/mL anti-interferon-g (BD Pharmingen, San Jose, CA, USA) and anti-CD3 and anti-CD28-coated beads (Invitrogen, Carlsbad, CA, USA).

For Treg induction, CD4^+^T cells were stimulated by 2 μg/ml anti-CD3 mAb, 4 μg/ml anti-CD28 mAbs, 5 ng/ml TGF-β and 2 ng/ml IL-2 for 48 h.

### Detection of Th17 and Treg cell frequencies by flow cytometry

PBMCs-derived lymphocytes were prepared by Percoll gradient centrifugation and then incubated with Cell Stimulation Cocktail (eBioscience, San Diego, CA, USA). For intracellular staining, single-cell suspensions were prepared, surface-stained with FITC-labeled anti-CD4 antibody (Miltenyi Biotec, Germany), and then fixed and permeabilized, stained with CD4-PE-Cy5 antibody (BD Biosciences, San Diego, CA, USA) for 30 min and labeled with either IL-17-PE or Foxp3-FITC antibodies (BD Biosciences). Flow cytometry was then performed to determine the percentage of Th17 and Treg cells after fixed with 1.5% paraformaldehyde as previously described (Wang 2017a).

### Cell transfection

CD4^+^ T cells isolated from PBMCs of OA patients were plated in 12-well plates. The culture medium was replaced by culture medium without antibiotics and 10% FBS one day prior to cell transfection. Until they were approximately 70% confluent, cells (1 × 10^6^) were transfected with miR-206 mimic, miR-206 inhibitor, pcDNA3.1-SOCS3, pcDNA3.1-Foxp3, siRNA specifically targeting SOCS3 (si-SOCS3), si-Foxp3, and scrambled negative controls (all purchased from Shanghai GenePharma, Shanghai, China) using HiPerFect Transfection Reagent (Qiagen, Hilden, Germany) at a concentration of 75 nM, before the induction of Th17 or Treg polarization. SOCS3 or Foxp3 expression vector was constructed by inserting the overall sequence of SOCS3 or Foxp3 into the pcDNA 3.1 vector. Transfection efficiency was assessed by real-time PCR.

### Luciferase reporter assay

Dual-luciferase reporter assays were employed to examine the interaction between miR-206 and SOCS3 or Foxp3 in CD4^+^T cells according to the manufacturer’s instructions. The wild-type (pmirGLO-SOCS3/Foxp3 Wt) and mutant-type (pmirGLO-SOCS3/Foxp3 Mut) luciferase reporter plasmids (Promega, Madison, WI, USA) were constructed according to the prediction results. The luciferase reporter plasmids and miR-206 mimic/mimic negative control (NC) were co-transfected into CD4^+^T cells. The luciferase activity was detected at 48 h posttransfection.

### Real-time PCR

After extracting total RNA with Trizol reagent, cDNA was obtained by First Strand cDNA Synthesis Kit (Roche Diagnostics, Indianapolis, IN, USA) following the manufacturer’s instructions. Real-time PCR was performed using SYBR Green PCR Kit (Takara Biochemicals, Kyoto, Japan). The thermocycling conditions used for qPCR were as follows: Initial denaturation at 95 °C for 3 min; followed by 40 cycles of 12 s at 95 °C and 40 s at 62 °C; the fluorescence signal was collected after 40 cycles. Relative fold changes in mRNA expression were calculated using the 2^−∆∆CT^ method (Livak and Schmittgen 2001). The relative expression of miR-206, SOCS3, Foxp3, STAT3, and RORγt were normalized to U6 and glyceraldehyde-3-phosphate dehydrogenase (GAPDH). Each sample was tested in triplicate for statistical analysis. The following primers were used in qRT-PCR: miR-206: 5'-GATTCGCCAAAGGAAATAGC-3' (forward), 5'-GTTACAAGGTCATCCAAGAC-3' (reverse); SOCS3: 5'-CCTGCGCCTCAAGACCTTC-3' (forward), 5'-GTCACTGCGCTCCAGTAGAA-3' (reverse); Foxp3: 5'-TTTCTGTCAGTCCACTTCACCA-3' (forward), 5'-CCAGCAGGTCTGAGGCTTTG-3' (reverse); STAT3: 5'-ACCCACTCCTTGCCAGTTGT-3' (forward), 5'-GGCCACTTGATCCCAGGTT-3' (reverse); RORγt: 5'-TGAGAAGGACAGGGAGCCAA-3' (forward), 5'-GAGAAGCTGAGTGCCATGCA-3' (reverse); U6: 5'-CTCGCTTCGGCAGCACA-3' (forward), 5'-AACGCTTCACGAATTTGCGT-3' (reverse); GAPDH: 5'-TGTGGGCATCAATGGATTTGG-3' (forward), 5'-ACACCATGTATTCCGGGTCAAT-3' (reverse).

### Western blot

Total protein was extracted from cultured CD4^+^T cells and PBMCs using RIPA lysis buffer (Beyotime Biotechnology, Shanghai, China), loaded on 10% sodium dodecyl sulfate polyacrylamide gel electrophoresis (SDS-PAGE) and blotted on polyvinylidene fluoride (PVDF) membranes. Following blocking with 5% skimmed milk for 2 h at room temperature, these membranes were incubated with primary antibodies against SOCS3 (#52,113), Foxp3 (#12,653), STAT3 (#12,640), RORγt (#16,540) (all from Cell Signaling Technology, Boston, MA, USA;1:1,000 dilution) overnight at 4˚C. The next day, membranes were incubated at room temperature for 2 h with FITC-labeled IgG secondary antibodies (#7074; 1:2,000 dilution; Cell Signaling Technology). The enhanced chemiluminescence reagent (Beckman Coulter, Brea, CA, USA) was used to detect the protein bands, and the protein expression levels were quantified using ImageJ software (version 1.6.0; National Institutes of Health, Bethesda, MD, USA).

### In vivo experiments

Adult male Sprague–Dawley rats weighing 300 ~ 350 g were averagely divided into several subgroups (n = 6 per group), namely, Sham, OA and OA + antagomir-206. To establish OA model, rats were given 0.1 g/kg ketamine hydrochloride for intraperitoneal anesthesia. Under the operating microscope, the medial collateral ligaments of the right knee, medial meniscus and the anterior and posterior cruciate ligaments were sequentially cut off. The sham-operated rats underwent the identical procedure except ligament resection. Rats in the OA + antagomir-206 group were treated with the intraarticular delivery of 800 pmol/g antagomir-206 (Shanghai GenePharma) 6 h prior to surgery. Finally, PBMCs were obtained after euthanasia at 6 weeks postoperatively. All animal procedures were approved by the Animal Care and Use Committee at Hubei Provincial Hospital of Traditional Chinese Medicine.

### Statistical analysis

All results were represented as mean ± standard deviation (SD) and analyzed using Graphpad Prism version 5.0 software (GraphPad, San Diego, CA, USA). Differences between two groups were analyzed by Student’s t-test. Comparisons between multiple groups were performed by one-way analysis of variance followed by Tukey's post hoc test. Pearson’s correlation analysis was performed to determine the correlation between the expression of miR-206 and Th17/Treg ratio in PBMCs of OA patients. *P* < 0.05 was considered to indicate a statistically significant difference.

## Results

### MiR-206 expression was correlated with Th17/Treg imbalance in patients with OA

**Fig. 1:**
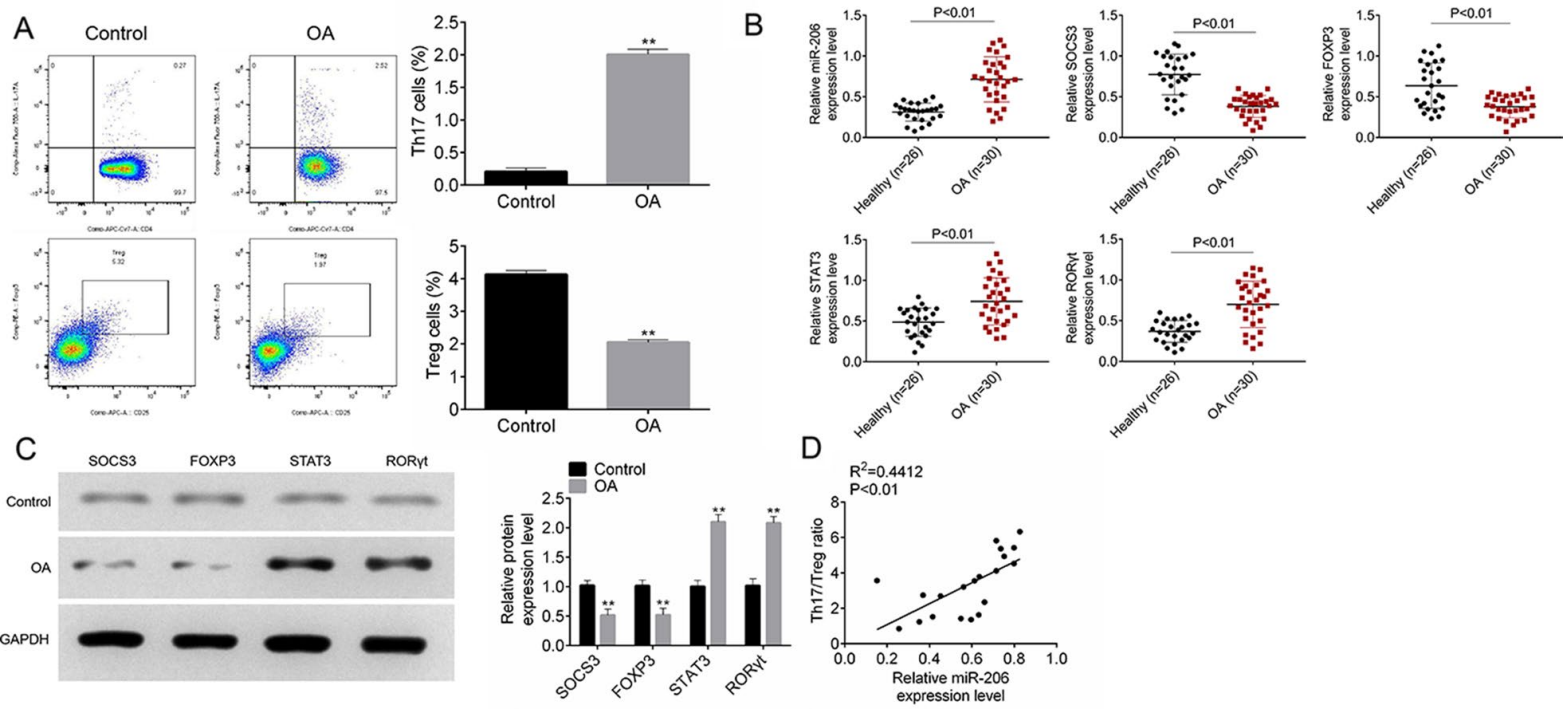
MiR-206 expression was correlated with Th17/Treg imbalance in patients with OA. The frequencies of Th17 and Treg cells (**A**), quantitative RT-PCR analyses of miR-206, SOCS3, Foxp3, STAT3, RORγt expression (**B**), and western blot analysis of SOCS3, Foxp3, STAT3 and RORγt protein levels (**C**) in PBMCs of OA patients and healthy controls; the correlation of miR-206 with Th17/Treg ratio in PBMCs of OA patients (**D**). All experiments were repeated three times. Data are expressed as mean ± SD. **P < 0.01

We investigated the frequencies of Th17 and Treg cells by flow cytometry analysis of CD4^+^ gated cells isolated from PBMCs of OA patients and healthy controls. Our results demonstrated that OA patients exhibited a higher frequency of Th17 cells, and a lower proportion of Treg cells compared with the control group (Fig. 1A, Additional file 1: Fig.S1; P < 0.01). To identify abnormally expressed miRNAs and specific regulators of Th17/Treg involved in OA development, we performed real-time PCR and western blot. Figure 1B, C showed that the expressions of miR-206, STAT3 and RORγt were significantly elevated, while SOCS3 and Foxp3 expression were markedly decreased in OA patients relative to the control group (5.12-fold upregulation for miR-206, 0.985-fold reduction for SOCS3, 0.973-fold reduction for Foxp3, 2.15-fold upregulation for STAT3, 2.25-fold upregulation for RORγt; all *P* < 0.01). As shown in Fig. 1D, the correlation between miR-206 expression and the frequencies of Th17 and Treg cells was analyzed by using Pearson’s correlation analysis, arriving at conclusions that the miR-206 expression was positively related with Th17/Treg ratio in PBMCs of OA patients RORgt (R^2^ = 0.4412, *P* < 0.01).

### *MiR-206 promoted the generation of Th17 cells *via* targeting SOCS3*

**Fig. 2:**
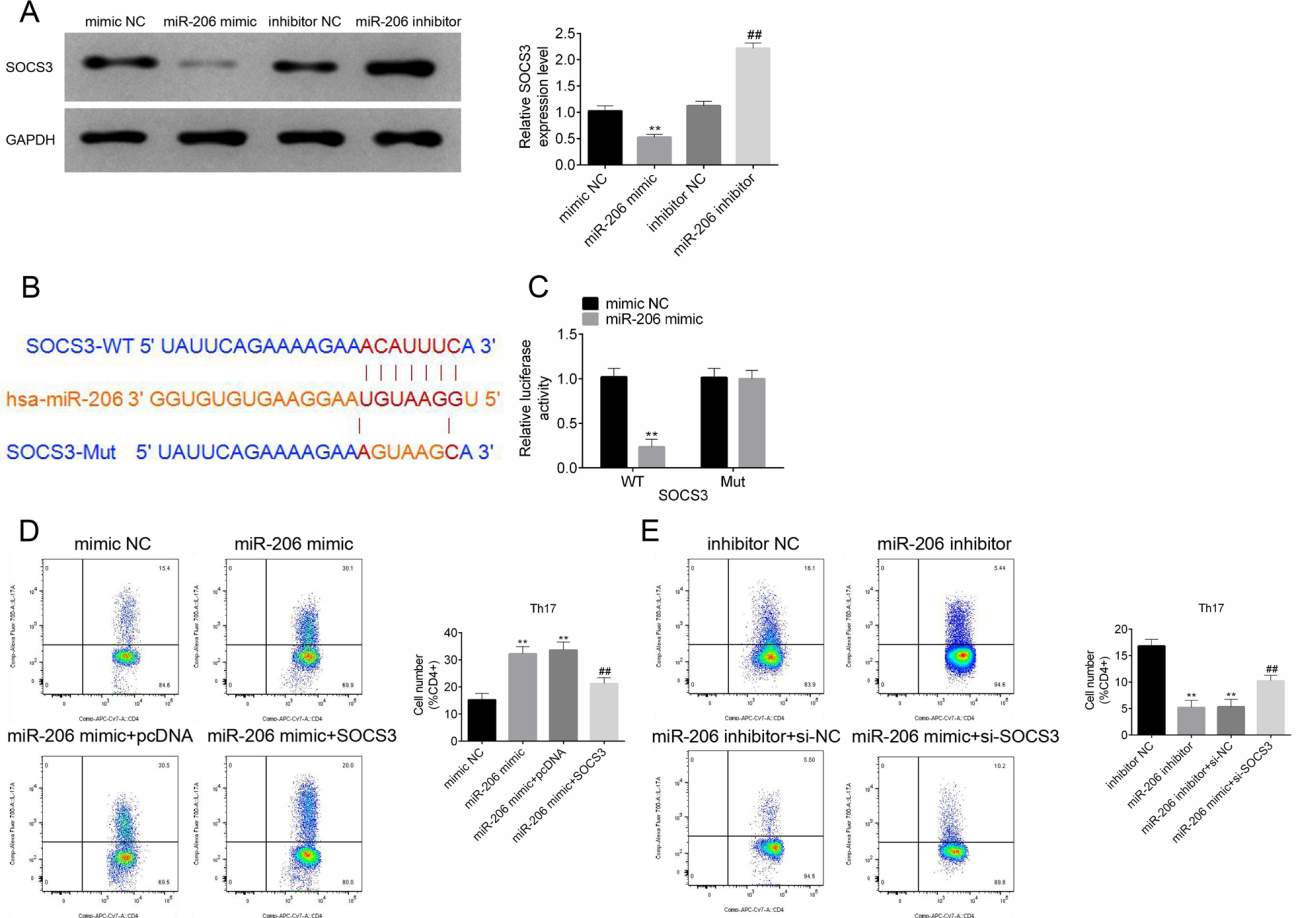
MiR-206 promoted the generation of Th17 cells via targeting SOCS3. **A** Expression levels of SOCS3 in CD4^+^T cells in response to miR-206 overexpression or knockdown; **B** Schematic illustration of miR-206 binding sites in SOCS3; **C** Luciferase assays of the indicated cells transfected with pmirGLO-SOCS3-WT/MUT reporters and miR-206 mimic/mimic NC; **D**, **E** The percentage of Th17 cells in CD4^+^ T cells in response to co-overexpression or co-knockdown of miR-206 and SOCS3. All experiments were repeated three times. Data are expressed as mean ± SD. **P < 0.01; ^##^*P* < 0.01

To identify the relationship between miR-206 and SOCS3, we transfected CD4^+^T cells with miR-206 mimic or inhibitor to overexpression or knockdown miR-206 expression (transfection efficiency was shown in Additional file 2: Fig.S2), and analyzed the expression of SOCS3 using western blotting. The results showed that miR-206 overexpression observably downregulated SOCS3 expression in CD4^+^T cells, while the knockdown of miR-206 led to the upregulation of SOCS3 protein levels (Fig. 2A; *P* < 0.01). Targetscan software (http://www.targetscan.org) was used to predict whether SOCS3 was a direct target of miR-206 (Fig. 2B; *P* < 0.01). Luciferase reporter assay was performed to confirm this interaction, and the results revealed that miR-206 mimic decreased the luciferase activities in SOCS3-WT co-transfected system (Fig. 2C; *P* < 0.01). The percentage of Th17 cells was significantly increased in miR-206 overexpressed CD4^+^ T cells, whereas, SOCS3 upregulation reversed this effect (Fig. 2D, Additional file 3: Fig.S3; *P* < 0.01). As expected, miR-206 inhibition and SOCS3 silencing reciprocally regulated the conversion of CD4^+^ T cells to Th17 cells (Fig. 2E, Additional file 4: Fig.S4; *P* < 0.01). The transfection efficiency of SOCS3 overexpression and knockdown was shown in Additional file 5: Fig.S5.

### MiR-206 inhibited the differentiation of Treg cells by decreasing Foxp3 expression

**Fig. 3:**
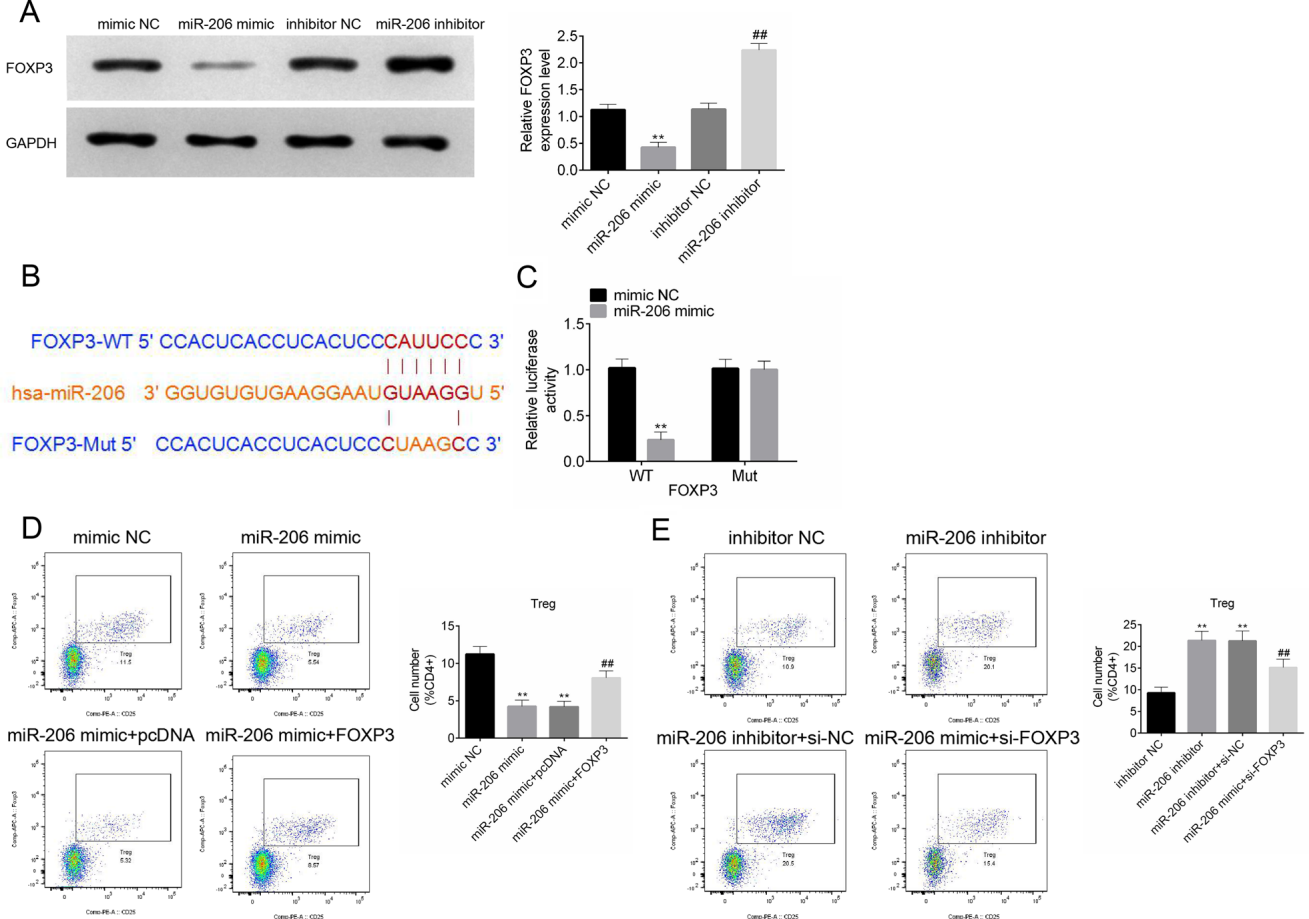
MiR-206 inhibited the differentiation of Treg cells by decreasing Foxp3 expression. **A** Expression levels of Foxp3 in CD4^+^T cells in response to miR-206 overexpression or knockdown. **B**, **C** Schematic illustration of miR-206 binding sites in Foxp3 and direct binding of miR-206 to Foxp3 by a dual luciferase reporter assay. **D**, **E** The percentage of Treg cells in CD4^+^ T cells in response to co-overexpression or co-knockdown of miR-206 and Foxp3. All experiments were repeated three times. Data are expressed as mean ± SD. **P < 0.01; ^##^*P* < 0.01

On the other hand, enforced expression of miR-206 could significantly downregulate the protein levels of Foxp3, while silencing miR-206 expression led to an opposite effect (Fig. 3A; *P* < 0.01). The 3’UTR region of Foxp3 contained binding sites for miR-206 (Fig. 3B). Moreover, the result of a luciferase reporter assay showed that co-transfection with pmirGLO-Foxp3-WT vector and miR-206 mimic significantly reduced luciferase reporter activity (Fig. 3C; *P* < 0.01). Overexpression of miR-206 decreased the frequency of Treg cells. Furthermore, Foxp3 overexpression antagonized the inhibitory effects of miR-206 on Treg cells (Fig. 3D, Additional file 6: Fig.S6; *P* < 0.01). The transfection efficiency of Foxp3 overexpression and knockdown was shown in Additional file 7: Fig.S7. Depletion of miR-206 expression significantly enhanced Treg cell differentiation as evidenced by increased percentage of Treg cells. Moreover, this pattern was reversed by inhibition of Foxp3 expression in CD4^+^ cells (Fig. 3E, Additional file 8: Fig.S8; *P* < 0.01).

### Inhibition of miR-206 expression restored the balance of Th17/Treg during OA pathogenesis

**Fig. 4:**
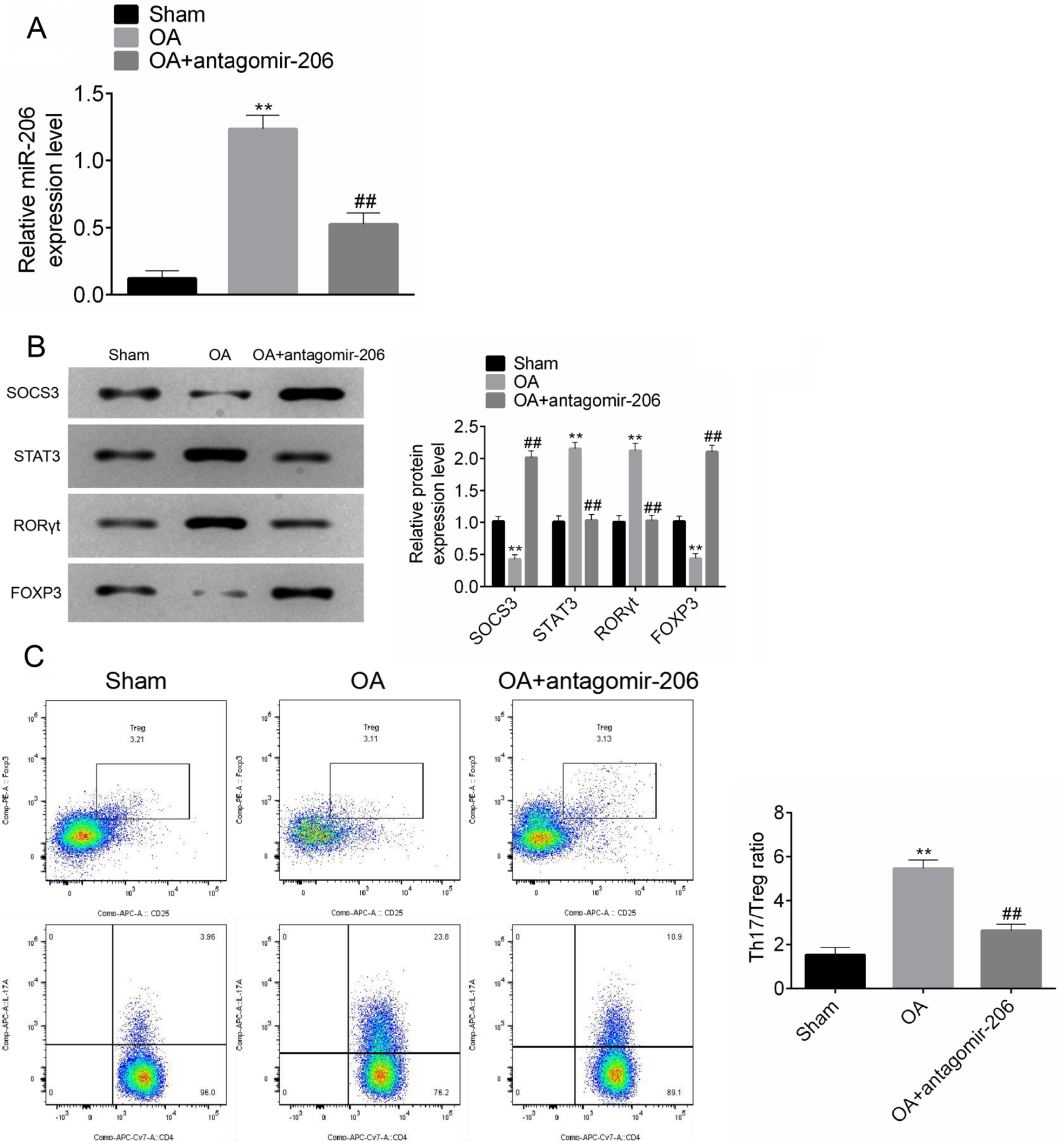
Inhibition of miR-206 expression restored the balance of Th17/Treg during OA pathogenesis. The expression of miR-206 (**A**), SOCS3, Foxp3, STAT3, RORγt (**B**), and Th17/Treg ratio (**C**) in rats with different groups. All experiments were repeated three times. Data are expressed as mean ± SD. **P < 0.01; ^##^*P* < 0.01

Based on the results of in vitro investigation, we speculated that knockdown of miR-206 may recover the Th17/Treg imbalance in OA in vivo. To test this hypothesis, the rats were treated with miR-206 antagomir prior to induction of OA model at 6 weeks after surgery. Compared with the sham group, miR-206 was highly expressed in PBMCs from OA rats; however, pretreatment with antagomir-206 downregulated the high expression of miR-206 caused by OA (Fig. 4A; *P* < 0.01). Knockdown of miR-206 by antagomir abolished OA-induced SOCS3 and Foxp3 downregualtion and STAT3 and RORγt upregulation (Fig. 4B; *P* < 0.01). The ratio of Th17/Treg cells was signally elevated in OA rats, which was dramatically reversed by antagomir-206 (Fig. 4C; *P* < 0.01).

## Discussion

Th17 and Treg cells are two diverse CD4^+^T-cell subsets with antagonistic effects and play important roles in maintaining the immune balance (Diller et al. 2016). Th17 cells are regarded as inflammatory helper T cells, which can specifically secrete IL-17 to mediate inflammation and autoimmunity. However, Treg cells express Foxp3 and produce TGF-β, IL-10, and IL-35, which mainly mediate immune tolerance or play an immunosuppressive role (Samid et al. 2016). Compelling evidence has delineated that Th17/Treg imbalance is implicated in a variety of chronic inflammatory diseases such as rheumatoid arthritis (Wang 2017b), septic arthritis (Dey and Bishayi 2017) and collagen-induced arthritis (Yang 2017). Previous clinical studies have confirmed that the levels of Th17 cells were significantly elevated in peripheral blood of OA patients (Askari 2016; Lurati 2015). Guo et al. observed decreased number of peripheral Treg cells in rats with OA, which was closely correlated to cardiopulmonary dysfunction (Guo 2015). Herein, OA patients showed higher frequencies of Th17 cells and upregulated STAT3 and RORγt mRNA and protein expression and lower Treg, and SOCS3 and Foxp3 mRNA and protein expression in PBMCs compared with healthy controls.

Recently, a growing body of evidence suggests that miRNAs are crucial for the pathogenesis of arthritic diseases through the modulation of Th17/Treg balance. For instance, Dong et al. implied that miR-21 was downregulated in CD4^+^ T cells of RA patients, and was negatively associated with Th17/Treg ratio (Dong 2014). Wu et al. indicated that miR-16 was adversely expressed in Th17 cells and Treg cells of active RA patients (Wu 2016). As demonstrated by previous studies, miR-206 was highly expressed in OA cartilage tissues, and miR-206 overexpression significantly inhibited the proliferation, promoted chondrocyte apoptosis, dramatically decreased Col2a1 and aggrecan, and increased Runx2 and MMP-13, indicating the involvement of miR-206 in cartilage degradation in OA (Ni et al. 2018). Recent studies showed that miR-206 silencing alleviated IL-1β-induced OA chondrocyte injury by inhibiting chondrocytes proliferation and promoting apoptosis (Liu 2018; Lu et al. 2020). In this report, OA patients showed increased miR-206 expression in PBMCs. A negative correlation between the percentages of Th17 cells and the expression of miR-206 was found in patients with dermatomyositis (Tang 2013). By Pearson’s correlation analysis, miR-206 expression in the PBMCs of OA patients was indicated to positively correlate with Th17/Treg ratio.

RORγt is a specific transcription regulator required for Th17 cell differentiation (Tae-Yoon 2014). SOCS3 is a negative regulator of STAT3/RORγt signaling pathway, which is essential for the inhibition of Th17 cell differentiation (Ding 2017). Previous studies demonstrated that SOCS3-mediated IL-6/STAT3 signaling pathway regulates multiple cytokine signaling pathways in autoimmune and infectious diseases (Shi 2019). Our previous study found that miR-206 directly targets SOCS3 and Foxp3 to facilitate Th17 differentiation and repress Treg differentiation in vitro*.* Intra-articular injection is a widely used method for local delivery of molecules into the knee joint (Wang 2019; Chen 2017), and treatment with antagomir-206 greatly alleviated the secretion levels of Th17-related cytokines and accelerated the production of Treg-related cytokines through affecting the expression of SOCS3 and Foxp3. The ability to modulate miRNA expression in vivo may provide an opportunity for the development of miR-206 as a therapeutic target for OA. However, we only validated that miR-206 inhibits SOCS3 or Foxp3 expression and thus leads to the disequilibrium of Th17 and Treg cells in OA. Given this fact, the clinical translation is limited. In a future study, ongoing clinical trials with recombinant SOCS3 or Foxp3 in human knee OA should be performed, thus offering an important perspective to support the therapeutic potential of miR-206 inhibition. Furthermore, the role of miR-206 in regulating chondrogenesis and cartilage remodeling needs to be further investigated.

## Conclusion

In summary, we concluded that miR-206 initiated the immune imbalance of Treg/Th17 in OA via targeting SOCS3 and Foxp3, suggesting that miR-206 may serve as a novel regulator of T cell differentiation. Inhibition of miR-206 provided a novel therapeutic target for the treatment of OA.

## Supplementary Information

**Additional file 1: Figure S1.** Representative flow cytometric analyses of the frequencies of Th17 and Treg cells in OA patients and healthy controls.

**Additional file 2: Figure S2.** The transfection efficiency of miR-206 overexpression and knockdown.

**Additional file 3: Figure S3.** Representative flow cytometric analyses of the frequencies of Th17 cells in CD4^+^ T cells in response to co-overexpression of miR-206 and SOCS3.

**Additional file 4: Figure S4.** Representative flow cytometric analyses of the frequencies of Th17 cells in CD4^+^ T cells in response to co-knockdown of miR-206 and SOCS3.

**Additional file 5: Figure S5.** The transfection efficiency of SOCS3 overexpression and knockdown.

**Additional file 6: Figure S6.** Representative flow cytometric analyses of the frequencies of Treg cells in CD4^+^ T cells in response to co-overexpression of miR-206 and Foxp3.

**Additional file 7: Figure S7.** The transfection efficiency of Foxp3 overexpression and knockdown.

**Additional file 8: Figure S8.** Representative flow cytometric analyses of the frequencies of Treg cells in CD4^+^ T cells in response to co-knockdown of miR-206 and Foxp3.

## Data Availability

The datasets used and/or analyzed during the current study are available from the corresponding author on reasonable request.
