# Supplementary figures and images for: MiR-206 regulates the Th17/Treg ratio during osteoarthritis
Source: Mol Med. 2021 Jun 19;27:64. doi: 10.1186/s10020-021-00315-1 (PMC8214293; doi:10.1186/s10020-021-00315-1)

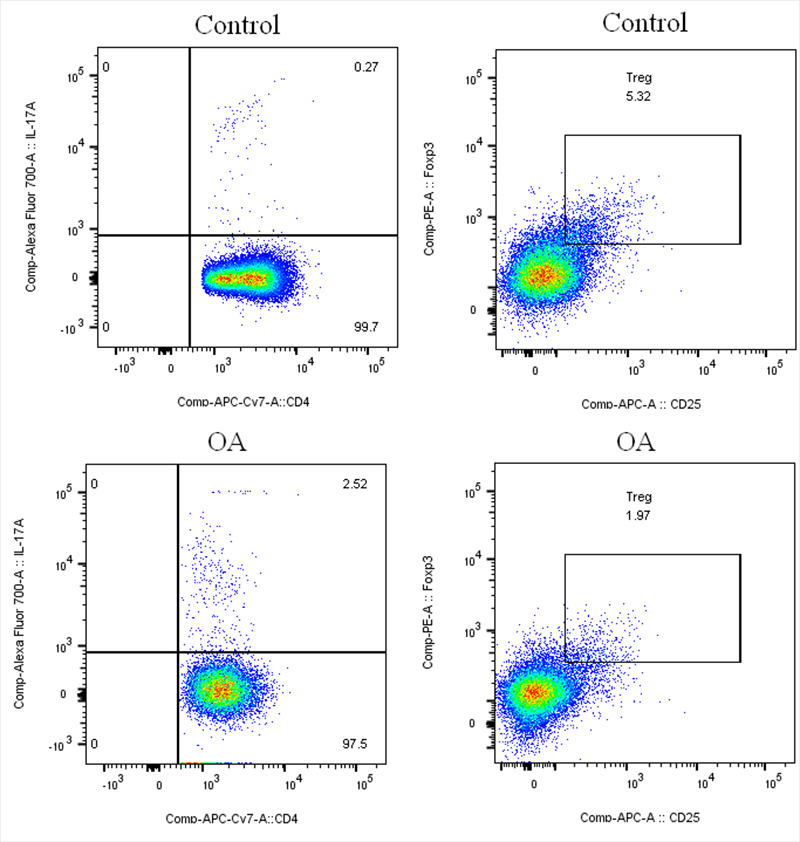

Supplement: Supplementary file 1 — Additional file 1: Figure S1. Representative flow cytometric analyses of the frequencies of Th17 and Treg cells in OA patients and healthy controls. [file 10020_2021_315_MOESM1_ESM.tif]

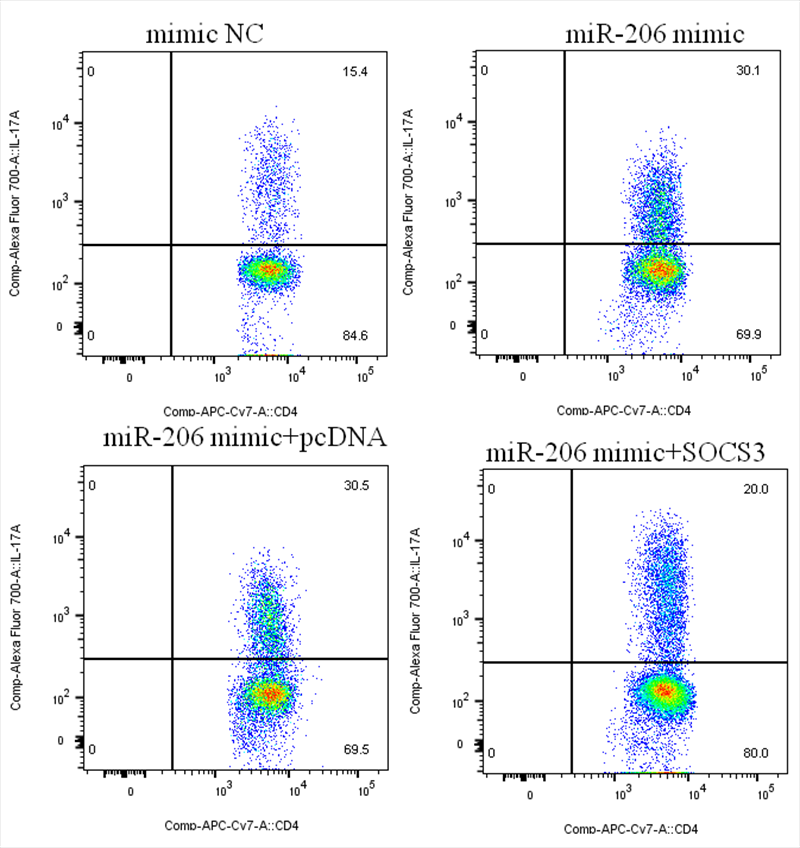

Supplement: Supplementary file 3 — Additional file 3: Figure S3. Representative flow cytometric analyses of the frequencies of Th17 cells in CD4+ T cells in response to co-overexpression of miR-206 and SOCS3. [file 10020_2021_315_MOESM3_ESM.tif]

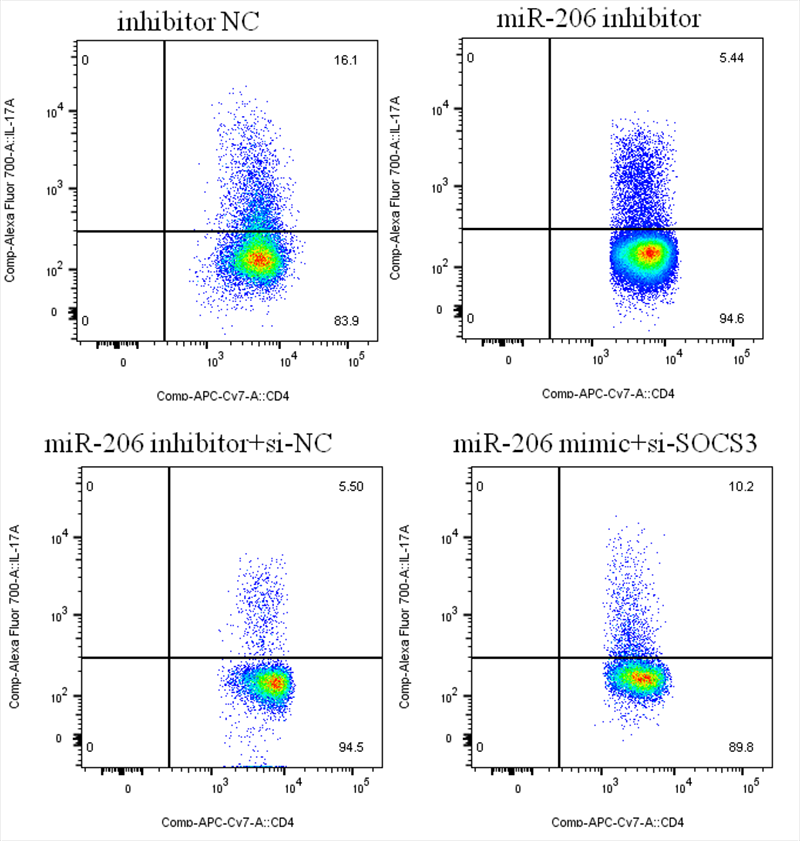

Supplement: Supplementary file 4 — Additional file 4: Figure S4. Representative flow cytometric analyses of the frequencies of Th17 cells in CD4+ T cells in response to co-knockdown of miR-206 and SOCS3. [file 10020_2021_315_MOESM4_ESM.tif]

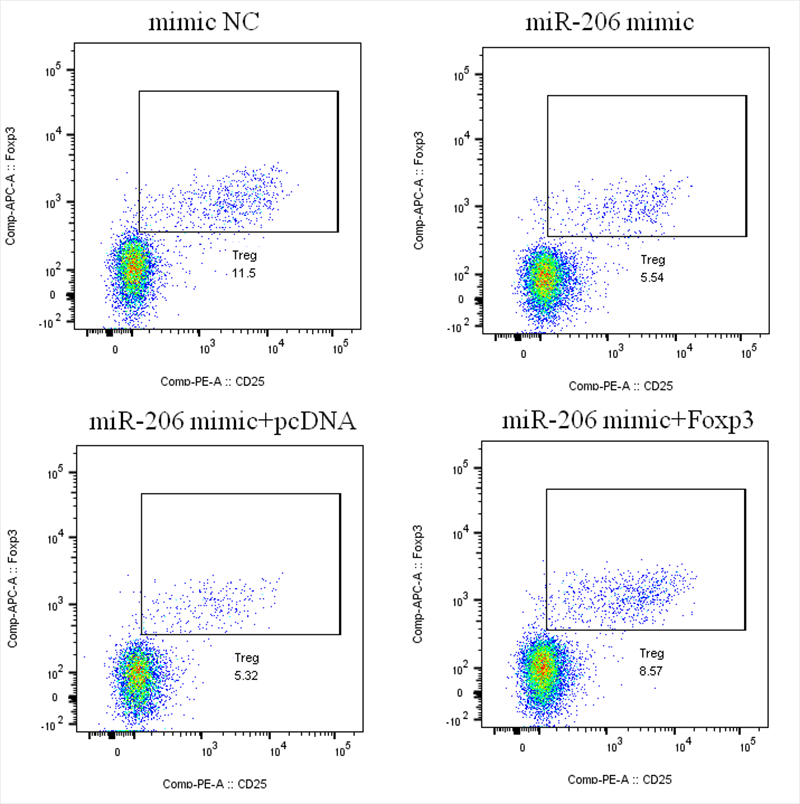

Supplement: Supplementary file 6 — Additional file 6: Figure S6. Representative flow cytometric analyses of the frequencies of Treg cells in CD4+ T cells in response to co-overexpression of miR-206 and Foxp3. [file 10020_2021_315_MOESM6_ESM.tif]

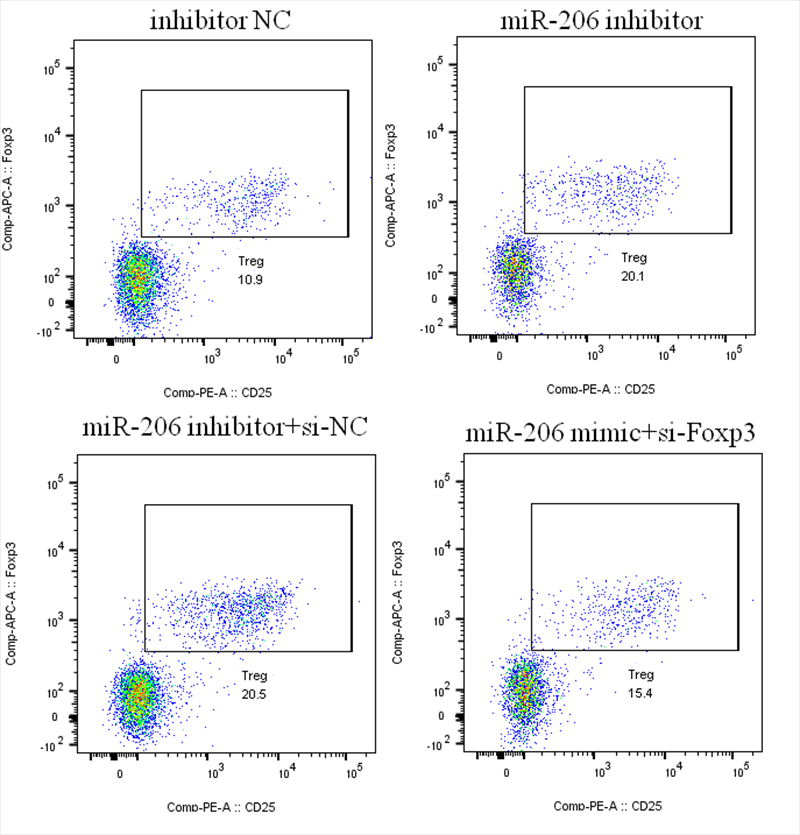

Supplement: Supplementary file 8 — Additional file 8: Figure S8. Representative flow cytometric analyses of the frequencies of Treg cells in CD4+ T cells in response to co-knockdown of miR-206 and Foxp3. [file 10020_2021_315_MOESM8_ESM.tif]
